# Intrinsically Connected: Therapeutically Targeting the Cathepsin Proteases and the Bcl-2 Family of Protein Substrates as Co-regulators of Apoptosis

**DOI:** 10.3390/ijms22094669

**Published:** 2021-04-28

**Authors:** Surinder M. Soond, Maria V. Kozhevnikova, Lyudmila V. Savvateeva, Paul A. Townsend, Andrey A. Zamyatnin

**Affiliations:** 1 Institute of Molecular Medicine, Sechenov First Moscow State Medical University, Trubetskaya str. 8-2, 119991 Moscow, Russia; ludmilaslv@yandex.ru; 2 Hospital Therapy Department No. 1, Sechenov First Moscow State Medical University, 6-1 Bolshaya Pirogovskaya str, 119991 Moscow, Russia; kozhevnikova-m@inbox.ru; 3 Division of Cancer Sciences and Manchester Cancer Research Centre, Faculty of Biology, Medicine and Health, University of Manchester, Manchester M20 4GJ, UK; paul.townsend@manchester.ac.uk; 4 Faculty of Health and Medical Sciences, University of Surrey, Guildford, Surrey GU2 7X, UK; 5 Belozersky Institute of Physico-Chemical Biology, Lomonosov Moscow State University, 119992 Moscow, Russia; 6 Department of Biotechnology, Sirius University of Science and Technology, 1 Olympic Ave, 354340 Sochi, Russia

**Keywords:** apoptosis, Bcl-2, BH3, extrinsic, intrinsic, MOMP, cell death, cancer, cathepsins

## Abstract

Taken with the growing importance of cathepsin-mediated substrate proteolysis in tumor biology and progression, the focus and emphasis placed on therapeutic design and development is coming into fruition. Underpinning this approach is the invariable progression from the direction of fully characterizing cathepsin protease members and their substrate targets, towards targeting such an interaction with tangible therapeutics. The two groups of such substrates that have gained much attention over the years are the pro- and anti- apoptotic protein intermediates from the extrinsic and intrinsic signaling arms of the apoptosis pathway. As proteins that are central to determining cellular fate, some of them present themselves as very favorable candidates for therapeutic targeting. However, considering that both anti- and pro- apoptotic signaling intermediates have been reported to be downstream substrates for certain activated cathepsin proteases, therapeutic targeting approaches based on greater selectivity do need to be given greater consideration. Herein, we review the relationships shared by the cathepsin proteases and the Bcl-2 homology domain proteins, in the context of how the topical approach of adopting ‘BH3-mimetics’ can be explored further in modulating the relationship between the anti- and pro- apoptotic signaling intermediates from the intrinsic apoptosis pathway and their upstream cathepsin protease regulators. Based on this, we highlight important future considerations for improved therapeutic design.

## 1. Introduction

Over the last 10 years, great strides have been taken in identifying cathepsin protease-specific substrates, which have revealed a number of interesting regulatory paradigms based on the growing pleiotropic nature of these enzymes. While they were originally isolated as lysosomal proteases involved in the proteolysis of intracellular proteins, their role in determining the fate of cells as active components arising from the lysosomal ‘suicide bag’ have seen their importance evolve [1,2]. At the molecular level, mechanistic insights into their substrate specificity have been slow to take shape as the cathepsin family of proteases is composed of a large number of members and subgroups, and which have similar yet distinct biochemical properties and substrate specificity [3,4]. Briefly, this protease family contains 15 members that can be further sub-divided into aspartic (D and E), serine (A and G), or cysteine cathepsin proteases (B, C, F, H, K, L, O, R, S, V, X/Z), and endo- and exo-peptidases [5]. Herein, the cysteine cathepsin proteases have received considerable attention based on their ability to remain catalytically active at relatively low and neutral pH [6,7]. Mechanistically, cathepsin proteases have been reported as tightly regulated at the protein level by their cognate inhibitors, the cystatins, but have also been reported as deregulated and overexpressed in a number of disease states such as cancer, and thus have a high level of therapeutic, diagnostic, and prognostic value [8]. Moreover, they are emerging to possess diversity in their subcellular compartmentalization, based on them reported to reside cytoplasmically, within the nucleus, with mitochondria, in addition to being localized within the extracellular compartment [5,9].

When taken with the ability of cathepsins to reside in the cytoplasm, the potential for them to cleave and modulate a number of biochemically significant signaling pathway intermediates central to determining cell viability has taken on heightened importance. One such process that the cathepsins have been identified to regulate is apoptosis (reviewed in [5,10]). While apoptosis was originally discovered as an important mechanism in cell fate and tissue development, it has emerged over time as a central regulatory mechanism in the development and progression of certain cancers [11], autoimmune diseases [12], and neurodegenerative disorders [13,14].

Generally, apoptosis is the end-point of two main regulatory pathways [15]. Firstly, the extrinsic pathway links extracellular death-inducing signals through receptor engagement, which culminates in caspase activation. For example, the binding of Fas or TNF-alpha ligands to their cognate receptors permit the formation of a death inducing signaling complex (DISC) and which signals through the activation of the cysteine-aspartic proteases caspase-8 or -10, to activate effector caspase-3, which can cleave a number of cellular macromolecules that initiate apoptosis [16]. Secondly, the intrinsic pathway, which is mainly regulated by the mitochondrion in response to cellular stress or growth factor deprivation [17,18], is responsible for the release of a number of mitochondrial-derived pro-apoptotic proteins. Here, this key regulatory step of mitochondrial outer membrane permeabilization (MOMP) can be mechanistically induced by a number of pro-apoptotic proteins from the B-cell lymphoma (Bcl-2) family, which mediate the formation of the mitochondrial permeability transition pore and have been the basis of many excellent studies that highlight significant value in therapeutically targeting this step [19]. The subsequently released mitochondrial proteins include Smac/DIABLO, apoptosis inducing factor (AIF), and cytochrome c [20,21], and form what is referred to as the ‘apoptosome complex’, which gives rise to the activation of caspases-9 and -3 [22,23,24]. The activation of the execution caspases -3, -6, and -7, leads to the activation of cytoplasmic CAD nucleases, which degrade nuclear lamin proteins, inhibit the DNA repair enzyme PARP, and which gives rise to the variety of morphological changes that are characteristic of apoptosis [25]. Lastly, externalization of phosphatidylserine at the apoptotic cell surface signals the recognition of apoptotic cells and their uptake by phagocytosis [26].

**Figure 1:**
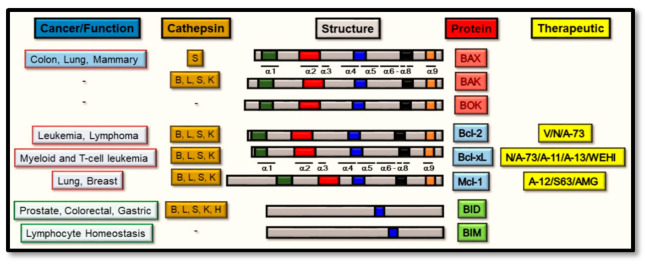
Schematic representation of the structural architecture of the Bcl-2 proteins, with the diseases (or function) and current therapeutics or cathepsin proteases associated with each of them. The three main groups of Bcl-2 family composed of pro-apoptotic, anti-apoptotic and BH3-only proteins are highlighted showing their structural domains as orange (trans-membrane domain), blue, black, red, and green boxes as the Bcl-2 homology (BH) domains 1–4 (respectively) along with the cathepsins that cleave them. The therapeutics active for the stated Bcl-2 proteins are V (Venetoclax), N (Navitoclax), A-73 (ABT-737), A-11 (A-1165463), A-13 (A-1331852), WEHI (WEHI-539), A-12 (A-1210477), S63 (S63845), and AMG (AMG176).

Throughout this process, and of central importance from a regulatory standpoint, is the expression of the Bcl-2 family of proteins [27], the members of which determine the sensitivity of cells to apoptosis through the intrinsic apoptosis pathway. Broadly, these proteins can be divided into two main functional groups: the pro-apoptotic and the anti-apoptotic proteins, and both of which constitute a family of proteins that are in excess of 18 members [28]. Alternatively, this Bcl-2 family can be subdivided structurally into three main groups, based upon the similarities between their Bcl-2 Homology (BH) domains [29,30]. Firstly, the pro-apoptotic protein group includes BAX, Bcl-2-associated protein X; BAK, Bcl-2 antagonist/killer; and BOK, Bcl-2 ovarian killer, and all structurally contain the four BH 1–4 domains and directly promote MOMP [31,32,33]. Secondly, members from the anti-apoptotic protein group all contain the four BH 1–4 domains: Bcl-2, B cell lymphoma-2; Bcl-xL, Bcl-2-related protein X; Bcl-w; Mcl-1; A1; or Bcl-B, which suppress MOMP by binding BAK or BAX directly. Thirdly, the pro-apoptotic BH3-only proteins group include Bim, Bcl-2 interacting mediator of cell death; Bad, Bcl-2 antagonist of cell death; BID, Bcl-2 interacting domain death agonist; Bmf, Bcl-2 modifying factor; Bik, Bcl-2 interacting killer-like protein; Noxa; Puma, p53-upregulated modulator of apoptosis and Hrk (Harakiri). As seen from Figure 1, all such members (except BID) contain one highly conserved BH3 domain, which is present in most pro-apoptotic proteins and may not always be absent in anti-apoptotic proteins [34]. However, the BH4 motif is present in all Bcl-2 anti-apoptotic proteins as a conserved domain [31,35].

Therapeutically, both extrinsic and intrinsic pathway intermediates have been the subject of intense scrutiny from a therapeutic targeting standpoint, helped and aided by the elucidation of the crystal structure of many signaling intermediates derived from these pathways [36]. Clearly, the rationale underlying most recent therapeutic development strategies have been directed at selectively manipulating the two arms of the apoptotic pathway with a view to harnessing the cells own molecular signaling machinery to mediate cell death [37], through a number of approaches including the design of ‘BH3-mimetics’ [38,39,40]. Functionally, mutagenesis of the BH3 domain (spanning α-helices 1–2, [41]) from activated BAX (or BAK) was unveiled to highlight its critical homo-oligomerization role during the induction of apoptosis [42]. As a domain that also binds the hydrophobic groove of the anti-apoptotic proteins (spanning α-helices 2–5, [43]), this interaction gives effect to the inhibitory properties of anti-apoptotic proteins such as Bcl-xL [44], thus preventing BAX activation and homo-oligomerization. Consequently, targeting this interaction site between BAX (or BAK) and the anti-apoptotic Bcl-2 protein, through the design of BH3-mimetics was unveiled to hold high therapeutic potential [45]. While this has some clear-cut relevance in cancer development and progression (where deregulated apoptotic pathway intermediates commonly prevail [46]), a complex picture is, however, emerging about how the proposed therapeutic modulation of these pathways may also be interconnected with other signaling cascades of relevance. This is of particular importance for the avoidance of therapeutic-mediated side effects [47].

**Figure 2:**
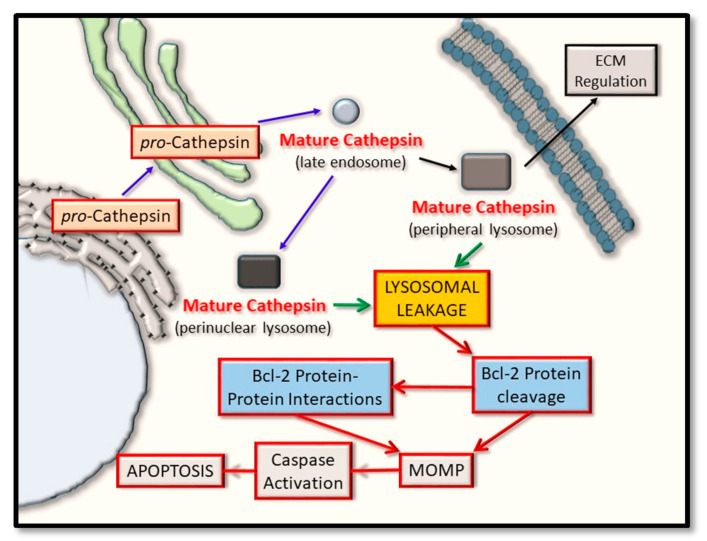
Cathepsin protein trafficking and intrinsic pathway activation for apoptosis. Cathepsin proteases are synthesized in their pro-inactive forms and mature through pro-domain removal, as they take on an endosomal and lysosomal localization. Through lysosomal leakage, they are released into the cytoplasm where they have the potential to proteolytically cleave certain Bcl-2 family protein members and thus alter their ability to form oligomers. Such events converge on regulating mitochondrial outer membrane permeabilization (MOMP) and have the effect of regulating caspase activation and cellular apoptosis.

Mechanistically, the cathepsin proteases have been linked to regulating both of the intrinsic and extrinsic signaling cascades, and which forms the basis of this review article by addressing what recent evidence supports their input into this regulatory step of apoptosis. Herein, we outline the rationale for therapeutically targeting certain cathepsin proteases, in the context of either selectively abrogating their activity for the breakdown (and destabilization of the pro-apoptotic Bcl-2 proteins), or their activity to be selectively maintained for the breakdown of the anti-apoptotic Bcl-2 proteins. Clearly, a favorable endpoint is effectively altering the balance of active pro-apoptotic Bcl-2 proteins levels in relation to their anti-apoptotic counterparts, with a view to tipping the balance of these proteins, in order to favor apoptosis in the context of killing cancer cells and halting tumor progression (Figure 2). Finally, we discuss what potential exists in furthering such findings to incorporate the simultaneous co-modulation of cathepsin protease activity and its relationship shared with pro- and anti- apoptotic Bcl-2 protein members, with a view to highlighting how the therapeutic design of BH3-mimetic can be utilized for greater effect.

## 2. Pro-Apoptotic BAX and BAK as Substrate Proteins for Cathepsin Proteases

Mammalian cells mainly encode three key pro-apoptotic proteins of importance, namely BAK, BAK and BOK, and each of which uniquely and directly induce MOMP based on their subcellular localization [33,48,49]. Whereas the BAX and BAK proteins are, respectively, cytoplasmic and mitochondrial [50,51]; BOK is associated with the ER and Golgi compartments [31,32,52,53,54,55]. The importance of each of these proteins during apoptosis originates from genetic studies strongly linking them to disease progression. For example, *BAX* gene frame-shift mutations can contribute to colon [56], lung, and T-cell acute lymphocytic leukemia cancer progression [57,58] and the loss of *BAX* expression can give rise to accelerated mammary tumor development in mice models [59]. From *BAX*-knockout mice studies, the observations of abnormal B- and T-cell numbers and compromised spermatogenesis [60] have been reported, in contrast to BAK-knockout studies, where no such developmental defects were observed [61]. Moreover, while mice with single-knockouts of both genes were susceptible to virus infection [62], double-knockouts were seen to be embryonic lethal [61,63,64]. Collectively, such findings highlight the distinct roles of BAX and BAK proteins during development while they may also share similar (or overlapping) roles at the molecular level during apoptosis. Unlike BAX and BAK [65,66], the role of the BOK protein, in mitochondrial cytochrome c release, in the absence of BAX and BAK expression, is less well-defined [67,68,69]. However, BOK-knockout mouse studies have highlighted the importance of the BOK protein in a developmental context, but its exclusive role in instigating developmental abnormalities appears to be redundant [70,71,72].

Based on such collectively strong evidence, whether such proteins are amenable to cathepsin-mediated digestion and deactivation has emerged as a key area of investigation, particularly in light of how deregulated cathepsin over-expression may also give cancer cells a survival and proliferative advantage during disease progression [5]. Mechanistically, a drop within the intracellular pH of cells favors enhanced cysteine-cathepsin protease activation [6,7], which are conditions that also induce BAX protein conformational changes, as seen in studies conducted in colon adenocarcinoma cells [73,74,75]. Collectively, such findings potentially offer a key (and novel) regulatory step, which may be exploited for therapeutic design with enhanced selectivity as a foresight. With this in mind, there have only been a limited number of published studies, which have fully addressed the ability of pro-apoptotic proteins to be cleaved by cathepsin proteases. For example, Cao et al. (2003) demonstrated that BAX could be cleaved by calpain at Asp-33 (giving p18BAX) and that this cleavage step could be abrogated in mammalian cells upon cathepsin inhibition to give a 25–35% reduction in drug-induced apoptosis [76]. Moreover, they showed that inhibition of p18BAX degradation enhanced apoptosis of A-549, U-937 and K-562 cells by 25–40%. Further evidence to support the importance of BAX Asp-33 cleavage came from studies, which concluded that a p21 BAX Asp-33-Ala mutation also disrupted BAX binding to the BID protein [77]. Similarly, Droga-Mazovec et al. (2008) reported the positive cleavage of BAK by cysteine cathepsins -B, -L, -S, and -K in vitro, whereas no cleavage of BAX by cathepsin S was observed [78]. While this could have been due to a number of reasons, from recent studies, our findings were supportive of BAX being directly cleaved to p18BAX in vitro by cathepsin S (at pH 5 or 7) and to complete digestion within intact mammalian cells. Moreover, the inhibition of cathepsin S had the effect of stabilizing p21BAX [79]. Based on the functional role of BAX and BAK up-regulating MOMP and apoptosis, many structural studies have revealed the potential to disrupt the interaction between the BAK BH3-domain with the hydrophobic groove of its cognate anti-apoptotic proteins, through designing BH3-mimetics and thus increasing the availability of monomeric BAK protein for MOMP and apoptosis induction [44].

While such BH3-mimetics do hold great therapeutic potential, their effects on cathepsin proteases, as key regulators for the cleavage of Bcl-2 family members and intrinsic pathway activation, remain largely unexplored. Similarly, the broader effects of cathepsin protease-directed inhibitors on the activation status of Bcl-2 family members, also remain poorly understood. This point is clearly highlighted through pepstatin A-mediated inhibition of cathepsin D, which delayed the onset of apoptosis, had the effect of altering the conformation of BAX, and which rendered it active independently of upstream BID cleavage-mediated activation [80]. Being mindful of such effects, in our recent study, we demonstrated BAX to be a novel and cathepsin S-targeted substrate. We also described the design of a novel cathepsin S-directed peptide inhibitor (CS-PEP1), based on a peptide sequence from the fungal papain protease inhibitor protein pit2, and which was identical to a sequence found within α-5 helix of the hydrophobic groove of anti-apoptotic Bcl-xL [79]. Mechanistically, CS-PEP1 was observed to act through the stabilization of BAX (through cathepsin S inhibition), or through possibly interfering with BAX-Bcl-xL protein binding. Collectively, such findings highlight a novel approach for inhibitor design targeted at BAX stabilization as a foresight, for this regulatory axis of the intrinsic apoptotic pathway. As proof of principle, CS-PEP1 enhanced apoptotic cell numbers at low doses, in good agreement with its intended purpose [79]. Such findings do suggest that pro-apoptotic proteins (such as BAX), along with their upstream regulator proteases, can be simultaneously targeted by a single therapeutic that can serve a dual purpose.

In summary, while a number of studies have looked at the intermolecular relationships between the cathepsins and Bcl-2 family of protein intermediates from the intrinsic pathway, one preferential cathepsin that directs BAX cleavage is cathepsin S (in vitro and in intact cells). Additionally, while other cysteine cathepsin proteases -B, -L, -S, and -K have been demonstrated to cleave BAK, no cathepsin proteases have been connected with BOK proteolysis. Moreover, progression towards therapeutically targeting this relationship, in order to spare enough pro-apoptotic monomeric protein for activation-mediated oligomerization, is indeed possible (as seen with the novel inhibitor CS-PEP1), and which offers a strong rationale for pursuing the BAK or BOK proteins as potential cathepsin substrates in a similar manner.

## 3. Anti-Apoptotic Bcl-2 Proteins as Substrates for the Cathepsin Proteases

The anti-apoptotic Bcl-2 proteins have also gained increasing importance for therapeutic targeting, from their abilities to drive cancer progression and the most extensive studies of which have described the targeting of Bcl-2, Bcl-xL, and Mcl-1 [81,82]. Mechanistically, while cancer cells can contain an abundance of pro-apoptotic Bcl-2 proteins, the amplification of anti-apoptotic Bcl-2 proteins can lead to BH3-only proteins being competitively depleted [83,84]. In the instance of the Bcl-2 anti-apoptosis protein, its amplification and overexpression can give rise to a number of hematological malignancies and solid tumors, such as lymphoma, prostate cancer, and small cell lung cancer [85,86,87]. Similarly, Bcl-xL can also drive tumor progression in the presence of Bcl-2 protein-directed therapeutic resistance [88,89,90], while Mcl-1 amplification has been reported in lung and breast cancers [88] and, thus, carries equal levels of importance. In all of these instances, the over-expression of these anti-apoptotic proteins have the effect of damaged cells surviving longer, thus permitting the accumulation of additional genetic lesions that can contribute to driving tumor progression [91,92]. In support, mouse knockout studies have yielded invaluable insights through delineating the dispensability of some anti-apoptotic proteins in cancer progression. For example, in Bcl-2^−/−^ mice, abnormal death of lymphocytes had been reported [91], while Bcl-xL^−/−^ mice possessed abnormalities in the demise of neurons and erythroid progenitor cells [92]. Additionally, Mcl-1^−/−^ mouse studies reported the death of early stage embryos and mice with conditional deletions experienced a rapid loss of mature lymphocytes or hematopoietic stem cells [93,94].

Based on the above evidence, the consequential role played by the cathepsin proteases during anti-apoptotic protein proteolysis has taken on significant importance. This was addressed by Droga-Mazovec et al. (2008), who reported the cleavage of proteins Bcl-2, Bcl-xL, and Mcl-1 upon the treatment of a variety of cell lines with the lysosomorphic agent, LeuLeuOMe [78]. More specifically, cathepsins -B, -L, -S, and -K were observed to cleave Bcl-2, Bcl-xL, and Mcl-1 proteins, using purified recombinant proteins in an in vitro cleavage assay at pH 7.2. As such, for substrates that are anti-apoptotic regulators, potential inhibition of cognate cathepsin proteases overexpressed during cancer development, can predictably have the effect of enhancing the levels of certain anti-apoptotic Bcl-2 sub-family members, and thus drive cancer progression. However, the significant (and simultaneous) benefits here may stem from preventing the proteolysis of pro-apoptotic Bcl-2 family members (such as BAX and BAK), particularly by cathepsins -B, -L, -K, and -S. Therefore, an ideal solution might involve the design of therapeutics that take on the properties of BH-3 mimetics, but have the flexibility and selectively, to inhibit cathepsin proteases against either of their pro-apoptotic or the anti-apoptotic protein substrates.

As very promising candidates for targeted cancer therapy, early studies identifying novel therapeutics targeting the Bcl-2 subfamily of anti-apoptosis proteins involved the screening of natural compounds and which yielded little success [95]. Through structural studies and rational drug-design approaches, a number of BH3-mimetics have been identified, and which promisingly act through binding the hydrophobic groove of the anti-apoptotic Bcl-2 protein, thus permitting enhanced monomeric BAK and BAX proteins to become activated through chemotherapeutic stimulation [83,96]. Such an approach led to the design of ABT-263 (or Navitoclax), a small inhibitor directed at Bcl-2, Bcl-xL, and BCl-w [84,97]. While it showed promising efficacy during phase I-II clinical trials for treating B-cell malignancies [97], its use was limited due to it inducing platelet-depleting effects [98,99,100,101]. Later, the cause of this was reported to be due to Bcl-xL inhibition having the effect of negatively modulating circulating platelets [102,103]. Nevertheless, a derivative of ABT-263 (called Venetoclax) did offer a successful treatment for patients with chronic lymphocytic leukemia (CLL), elapsed or refractory CLL and acute myeloid leukemia (AML) [104,105,106]. While this agent was largely ineffective for most solid tumors and chemotherapeutic resistance was common [107], it was evaluated as a useful therapeutic for treating thrombocytopenia [89]. This also formed the basis for the development of derivatives such as WEHI-539, A1155463, and A-1331852 [108,109,110], and from which, A-1331852 was reported as the first successful antagonist for Bcl-xL targeting. In the context of Mcl-1 inhibition, ABT-737, Venetoclax (or Navitoclax) [111,112] were evaluated as being largely ineffective therapeutics, but nevertheless highlights the specificity and exclusivity with which they can target certain anti-apoptotic targets [113,114]. Collectively, while such approaches do indeed highlight the power of BH3-mimetics, as with most therapeutics, their further development may be a necessity as therapeutic resistance and side effects can still present significant hurdles.

As seen from our recent studies outlining an alternative approach for developing a peptide inhibitor to target cathepsin S-specific Bcl-2 family intermediates of the intrinsic pathway, effective peptide efficacy (and specificity) may also be guided by the intracellular pH of cancer cells, as a key-determining factor. In this context, we observed that cathepsin S could cleave Bcl-xL in vitro better at pH 7 than at pH 5 and that this reaction could be inhibited better at pH 7 (than at pH 5), by the novel peptide inhibitor CS-PEP1. Such a pH-sensitive cleavage reaction appeared fortuitously to favor the inhibition of Bcl-xL cleavage (by CS-PEP1) at a relatively higher pH, in relation to the lower pH that can be prevalent during tumor development and apoptosis. Favorably, cathepsin S-mediated cleavage of BAX (and the inhibition of this reaction by CS-PEP1) was observed to be effective at both pH 5 and 7 [79]. Collectively, such findings offer an alternative approach in targeting this inhibitory axis of the intrinsic pathway for apoptosis with greater flexibility, through designing a more selective therapeutic that is based upon combining the principles of BH3-mimetics with the classical approach of cathepsin S-directed allosteric inhibition [79]. Importantly, the inhibition of cathepsin-mediated BAX (or possibly BAK) and Bcl-xL cleavage, may also be modulated further in a pH-dependent manner by such a therapeutic, thus permitting the favorable cleavage of an anti-apoptotic protein better than its pro-apoptotic counterpart. 

## 4. BH3-Only Domain Proteins as Substrates for the Cathepsin Proteases

From the family of multiple BH3-only proteins, the two most characterized members, in the context of being cleaved by cathepsin proteases, are BID and Bim [78,115,116]. While both proteins have evolved to sense cellular stress and strongly participate in the initiation of MOMP [117], like other members of this family they can be further classified as direct activators or sensitizers of BAX- or BAK-directed MOMP, based upon whether they bind anti-apoptotic proteins or displace direct activators of them [118]. Mechanistically, they may have distinct or partially overlapping binding preferences for the anti-apoptotic Bcl-2 protein [119] and can also act upstream of BAX or BAK, based on expression studies in BAX- or BAK- knockout fibroblast cells [120].

Functionally, the main role of BID appears to link the death receptor pathway to MOMP [121,122], following its cleavage by caspase-8 [123] or caspase-3 [124]. Through this step, it can drive the translocation and insertion of BAX into the outer mitochondrial membrane [125], eventually leading to BAX- (or even BAK-) dependent MOMP [126]. The interplay between cathepsin proteases and BID cleavage-mediated activation of MOMP is also strongly supported by mouse-knockout studies. Here, tissue extracts from BID^−/−^ mice failed to release mitochondrial cytochrome c upon their treatment with lysosomal extracts (in comparison to tissue extracts from normal mice), thus linking lysosomal-derived, cathepsin-mediated cleavage (and the activation) of BID, with MOMP [127,128]. Moreover, a similar effect was also evidenced upon the lysosomal leakage of cathepsins and upon cathepsin inhibition in human neutrophil cells [129]. From using such approaches, the cathepsin-mediated BID cleavage site had also been mapped to Arg-65 or Arg-71, and which can be cleaved by cathepsins -B, -H, -L, -S, and -K [115].

In confirming such observations, from our recent studies, purified BID could be cleaved by cathepsin S in vitro or in mammalian cells, and this cleavage effect could be reversed upon the co-expression of BID with catalytically-null cathepsin S [79]. While such findings do suggest a pro-survival effect for cathepsin S and BID co-expression, this must be viewed simultaneously with cathepsin S giving rise to a p18 cleaved form of BAX, and which has been shown to be of greater potency as an inducer of MOMP (in relation to p21 BAX) [76]. Nevertheless, when taken with cathepsin S as being a key protease for complete BAX cleavage, this highlights a novel paradigm that supports the idea that cathepsin S can simultaneously modulate extrinsic apoptotic signals (through BID cleavage) and intrinsic apoptotic signals through (BAX cleavage). This may also be understood as a molecular regulatory axis that may be amenable to therapeutic intervention, in order to permit the selective inhibition of either the extrinsic- or intrinsic apoptosis pathways, and indeed warrants further exploration [79].

In the instance of the Bim protein (Bcl-2 interacting mediator of cell death), its importance in cell death is highlighted through its negative selection of B- and T-cell precursors and regulation of peripheral lymphocyte homeostasis [30]. In support, Bim deficiency causes, 1) hyperplasia, which facilitates tumorigenesis, as seen from *c-myc* transgenic mice developing B-cell leukemia [130]; or, 2) tumorigenic outgrowth of p53- and E1A-transformed mouse baby kidney-derived epithelial cells [131]. The significance of such reported findings are also supported by the development of cell-based models utilizing Bim-directed siRNA knockdown studies [132,133,134,135]. Consequently, the role of Bim in tumorigenesis is taking on greater importance, as it is emerging as a viable target for cancer therapy through the design of BH3-mimetics [136].

As in the case of BAX and BAK, targeting the cathepsin(s) responsible for BH3-only protein degradation may hold good therapeutic value. In this context very little is known about how currently available anti-cathepsin therapeutics affect the activation (or proteolysis) of many other BH3-only proteins, including Bim.

## 5. Cathepsin and the Bcl-2 Proteins: Targeted Therapeutic Development

While a number of validated therapeutics directed at the Bcl-2 anti-apoptotic proteins have been described herein, further questions do arise to address what effects such therapeutics may have on the activity of upstream modulators, such as the cathepsin proteases. For example, during the inhibition of BAX-Bcl-2 protein binding, are BAX (or Bcl-2) protein molecules presented as good substrates for their cognate cathepsin proteases in the presence of BH3-mimetics? Based on recent developments (reported herein), this could indeed have an impact on the overall net stability of certain Bcl-2 proteins and how well they may fulfill their native roles as key apoptotic regulators.

**Figure 3:**
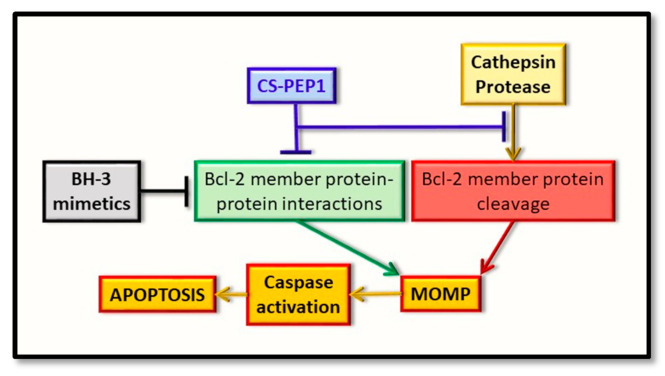
Representation of the early intrinsic arm of the apoptotic pathway, highlighting the key points identified for therapeutic intervention. Whereas BH3-mimetics (black box) can interfere with the pro-apoptotic BH3-domain interaction with the hydrophobic groove of the anti-apoptotic protein (green box), the novel inhibitor (CS-PEP1, blue box) can interfere with cathepsin S-mediated cleavage of the BAX protein (red box) while also potentially interfering with the respective BH3 domain-hydrophobic groove of BAX with Bcl-xL (green box). Consequently, BAX protein can be stabilized in a manner where it can be readily activated for the induction of MOMP and thus enhance apoptosis (orange boxes).

As a novel and alternative ‘semi-rational’ approach for therapeutic development, we have defined a peptide therapeutic based on its ability to potentially disrupt the BAX BH3-Bcl-xL hydrophobic groove interaction, while at the same time taking on properties of an allosteric inhibitor directed at the catalytic activity of cathepsin S. Of relevance, may also be the subcellular compartmentalization shared by the cathepsin and Bcl-2 proteins, and the penetrability of any arising therapeutics. In this context, while targeting the lysosome may be achieved with relative ease, other extra-lysosomal compartments, such as the unidentified and membranous perinuclear compartment in which enriched cathepsin S and BAX-derived proteins have been uniquely seen to co-localize (and for which we refer to as the ‘catheptasome’, until further characterization) may present additional challenges [79]. This is a key factor in any therapeutic targeting strategy and is one that warrants further consideration, in this instance. Nevertheless, all of the above approaches do indeed offer an alternative to therapeutic design and assessment, through their potential to target the regulatory effects of the cathepsin proteases as upstream regulators of the Bcl-2 family of proteins (Figure 3).

## 6. Future Directions

While one aspect of good therapeutic design directed at the Bcl-2 proteins appears to have taken shape and is yielding tangible effects against tumor progression and cancer in the form of BH3-mimetics [137], this review article does highlight a new avenue that could be explored as an alternative and more inclusive therapeutic design strategy. In light of the cathepsin proteases having such a notable impact as upstream regulators for the Bcl-2 family members of the extrinsic- and intrinsic- pathway of apoptosis, their incorporation into effective cancer therapeutic design strategies is timely. 

From our recent findings, one possible novel approach that could fulfill this is through identifying peptide sequences present in the cognate cathepsin protease inhibitor proteins (such as the cystatins), with a view to evaluating their abilities in modulating the pro- and anti-apoptotic protein interactions that the BH3-mimetics are designed to disrupt. Moreover, such therapeutics may also have the potential to interfere negatively with the cathepsin protease activity directed at certain pro-apoptotic protein members from the intrinsic apoptosis pathway. The net effect of such an approach may have greater benefits attached to them as such efforts may yield the development of a new generation of therapeutics that offer greater efficacy and selectivity. With this in mind, the resulting therapeutics may also have greater tolerance to operate at a fluctuating intracellular pH based upon the broad range at which the cysteine cathepsin protease are active, either within the tumor microenvironment or intracellularly in cells that have been chemotherapeutically treated. However, what effects such pH changes may have on cathepsin protease-substrate specificity, based upon substrate protein conformational changes and alterations in their sub-cellular localization, are also factors that need to be given greater consideration while defining the efficacy of any therapeutics arising from this novel approach.

## Abbreviations

BH domain (Bcl-2 homology domain); BAX (Bcl-2-associated protein X); BAK (Bcl-2 antagonist/killer); BOK (Bcl-2 ovarian killer); Bcl-2 (B cell lymphoma-2); Bcl-xL (B cell lymphoma extra-large); Bim (Bcl-2 interacting mediator of cell death); Bid (Bcl-2 interacting domain death agonist); Mcl-1 (myeloid cell leukemia-1 protein); MOMP (Mitochondrial Outer Membrane Permeabilization); Puma (p53-upregulated modulator of apoptosis); CAD (caspase-activated DNAase); PARP (poly ADP-ribose polymerase).
